# Sphingosine kinase 1 is overexpressed and promotes adrenocortical carcinoma progression

**DOI:** 10.18632/oncotarget.6564

**Published:** 2015-12-11

**Authors:** Yunze Xu, Baijun Dong, Jiwei Huang, Wen Kong, Wei Xue, Yu Zhu, Jin Zhang, Yiran Huang

**Affiliations:** ^1^ Department of Urology, Ren Ji Hospital, School of Medicine, Shanghai Jiaotong University, Shanghai, China; ^2^ Department of Urology, Ruijin Hospital, School of Medicine, Shanghai Jiaotong University, Shanghai, China

**Keywords:** adrenocortical carcinoma, sphingosine kinase 1, progression, prognosis, FTY720

## Abstract

Adrenocortical carcinoma (ACC) is a rare endocrine tumor with a very poor prognosis. Sphingosine kinase 1 (SphK1), an oncogenic kinase, has previously been found to be upregulated in various cancers. However, the role of the SphK1 in ACC has not been investigated. In this study, SphK1 mRNA and protein expression levels as well as clinicopathological significance were evaluated in ACC samples. *In vitro* siRNA knockdown of SphK1 in two ACC cell lines (H295R and SW13) was used to determine its effect on cellular proliferation and invasion. In addition, we further evaluated the effect of SphK1 antagonist fingolimod (FTY720) in ACC *in vitro* and *in vivo*, as a single agent or in combination with mitotane, and attempted to explore its anticarcinogenic mechanisms. Our results show a significant over-expression of SphK1 mRNA and protein expression in the carcinomas compared with adenomas (*P* < 0.01 for all comparisons). Functionally, konckdown of SphK1 gene expression in ACC cell lines significantly decreased cell proliferation and invasion. FTY720 could result in a decreased cell proliferation and induction of apoptosis, and the combination of mitotane and FTY720 resulted in a greater anti-proliferative effect over single agent treatment in SW13 cells. Furthermore, FTY720 could markedly inhibit tumor growth in ACC xenografts. SphK1 expression is functionally associated to cellular proliferation, apoptosis, invasion and mitotane sensitivity of ACC. Our data suggest that SphK1 might be a potential therapeutic target for the treatment of ACC.

## INTRODUCTION

Adrenocortical carcinoma (ACC) is a rare but highly aggressive endocrine malignancy with a 5-year overall survival of around 40% [1–2]. Therapeutic options for these patients are scarce, radical surgery resection remains the only potentially curative option for ACC until recently; however, patients often present with advanced localized invasion or initially distant metastases, limiting the opportunity for surgical resection. Despite the combination of cytotoxic drug and mitotane, the overall prognosis is still very poor in cases of advanced ACC [3]. Thus, there is still a critical need for the development of new treatment strategies for patients with ACC.

Sphingosine kinase 1 (SphK1), the rate-limiting enzyme of sphingosine 1 phosphate (S1P) synthesis, closely regulates the ceramide/sphingosine-S1P rheostat [4]. S1P is a bioactive lipid with oncogenic functions that promoting tumor cell growth and invasion. SphK1 has received increased attention during the past years [5–6]. SphK1 is overexpressed in various cancers, and the upregulation of SphK1 is associated with poor prognosis in many types of human cancers. Consistently, interference with SphK1 activity by dominant-negative mutants or competitive inhibitors, as well as inhibition of S1P by mAbs or S1P receptors antagonists, could significantly reduce cell proliferation, angiogenesis, and invasion, and increased apoptosis in some cancer cell lines [7–8].

Moreover, recent studies showed that SphK1 may be involved in resistance to both chemotherapeutics and targeted agents, suggesting that inhibitors of SphK1 in combination with chemotherapeutic treatments can synergistically act to induce tumor cell death [9–10]. However, no data are currently available on the efficacy of inhibitors of SphK1 in ACC, and the role of SphK1 in ACC progression remains unknown. In this study, we report for the first time characterization of SphK1 expression in human ACC tissues and their correlation with clinicopathologic factors. In addition, we further evaluated the effects of anti-SphK1 therapies [anti-SphK1-based small interfering RNA (siRNA) and pharmacologic inhibitior FTY720] in ACC cell lines, as a single agent or in combination with mitotane, and attempted to explore its anticarcinogenic mechanisms.

## RESULTS

### Upregulation of SphK1 expression in ACC

**Figure 1:**
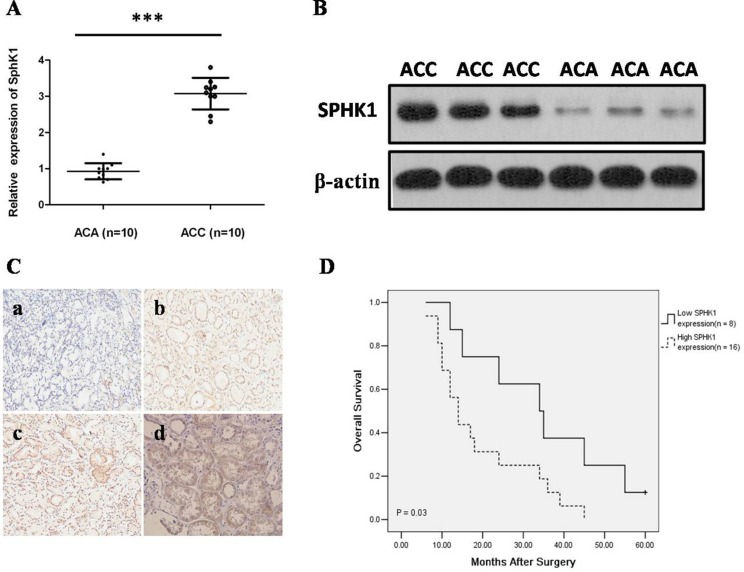
Expression of Sphk1 is elevated in ACCs (**A**) SphK1 mRNA expression analyzed by real-time RT-PCR in ACA and ACC tissues. SphK1 mRNA expression levels were normalized to GAPDH mRNA expression. SphK1 mRNA was determined to be at least three folds higher in the ACC tissues compared to ACA tissues ***P < 0.001; (**B**) Western blot analysis of SphK1 and sphingosine-1-phosphate Receptor 1 (S1P1R) protein in primary ACA and ACC tissues. Expression levels were normalized with β-actin; (**C**) Representative images from SphK1 immunohistochemistry in ACA and ACC tissues (20 × magnification). a) The expression of SphK1 was negative in ACA tissues; b) The expression of SphK1 was weakly positive staining in ACC with ENSAT 1; c) The expression of SphK1 was moderate positive staining in ACC with ENSAT 2; d) The expression of SphK1 was strong positive staining in ACC with ENSAT 3; (**D**) The association between SphK1 expression levels in tumors and overall survival in ACC patients using IHC score. Patients with high expression of SphK1 display a significant shorter overall survival after surgery. Kaplan-Meier method and log-rank test were used to evaluate overall survival and compare the differences between the two groups, *P* = 0.03.

Tumor characteristics and clinical features of 46 patients were summarized in Table 1. To determine the clinical and functional relevance of SphK1 in ACC, we initially assess the expression of SphK1 by western blot analysis and quantitative RT-PCR assay in 10 ACC and 10 ACA freshly frozen tissue samples. Our results showed that SphK1 was significantly upregulated at both mRNA and protein levels in ACC samples when compared with ACA samples (*P* < 0.001; Figure 1A and 1B). Then, we examined SphK1 protein expression in 24 ACC and 22 ACA pathology specimens using immunohistochemical staining. Representative photomicrographs of SphK1 immunostaining are shown in Figure 1C. We observed that 79.17% (16/24) of the cancer tissue samples showed high SphK1 expression, however, only 18.18% (4/22) of the benign group showed high SphK1 expression, the difference of SphK1 expression between ACAs and ACCs was statistically significant (*P* < 0.001). Immunoreactivity for SphK1 was mainly localized in the cytoplasm of cancer cells, which is consistent with previous studies on SphK1 expression in other types of cancer.

**Table 1:** Tumor characteristics and clinical features of patients in our study

Clinical characteristic	ACCs	ACAs
**Total number of tumors/patients**	24/24	22/22
**Gender**		
**Male**	10	8
**Female**	14	14
**Age at presentation (year) mean (range)**	51.17 ± 8.19 (38–71)	43.50 ± 9.55 (28–60)
**Tumor location**		
**Left adrenal**	11	9
**Right adrenal**	13	13
**Bilateral**	0	0
**Primary tumor size**		
**< 5.0 cm**	7	19
**> 5.0 cm**	17	3
**Mean diameter (cm)**	7.59 ± 2.52	3.45 ± 1.41 (P = 0.023)
**ENSAT Stage**		
**1**	5	
**2**	7	
**3**	10	
**4**	2	
**Previous therapies**		
**Complete resection**	24	22
**Adjuvant mitotane after surgery**	10	0
**Mitotane combined with streptozotocin or EDP**	0	0

Moreover, SphK1 expression strongly correlated with ENSAT 3–4 (*P* = 0.023) and tumor size (*P* = 0.001), however, the expression level of SphK1 did not differ significantly by age, gender, and side (Table 2). As shown in Figure 1D, the length of survival time was significantly different between patients with low and high SphK1 expression (*P* = 0.03), with the low SphK1 group having a longer overall survival time. Our data revealed that overexpression of SphK1 may play a biological role in ACC.

**Table 2:** Correlation between SphK1 expression and clinicopathologic characteristics of ACC patients

	Ovearall (*n* = 24)	Sphk1 Staining Low (*n* = 8)	High (*n* = 16)	*P*
**Age, y**				
**≥ 50**	13	4	9	1.000
**< 50**	11	4	7	
**Gender**				
**Female**	14	5	11	1.000
**Man**	10	3	5	
**Side**				
**Right adrenal**	13	3	10	0.390
**Left adrenal**	11	5	6	
**Tumor size**				
**≥ 5.0 cm**	17	2	15	0.001
**< 5.0 cm**	7	6	1	
**ENSAT Stage**				0.023
**1**	5	4	1	
**2**	7	3	4	
**3**	10	1	9	
**4**	2	0	2	
**Overall survival median (months)**	17.5	34.5	14	0.03

### Knockdown of Sphk1 suppresses cell proliferation and invasion in ACC cell lines

**Figure 2:**
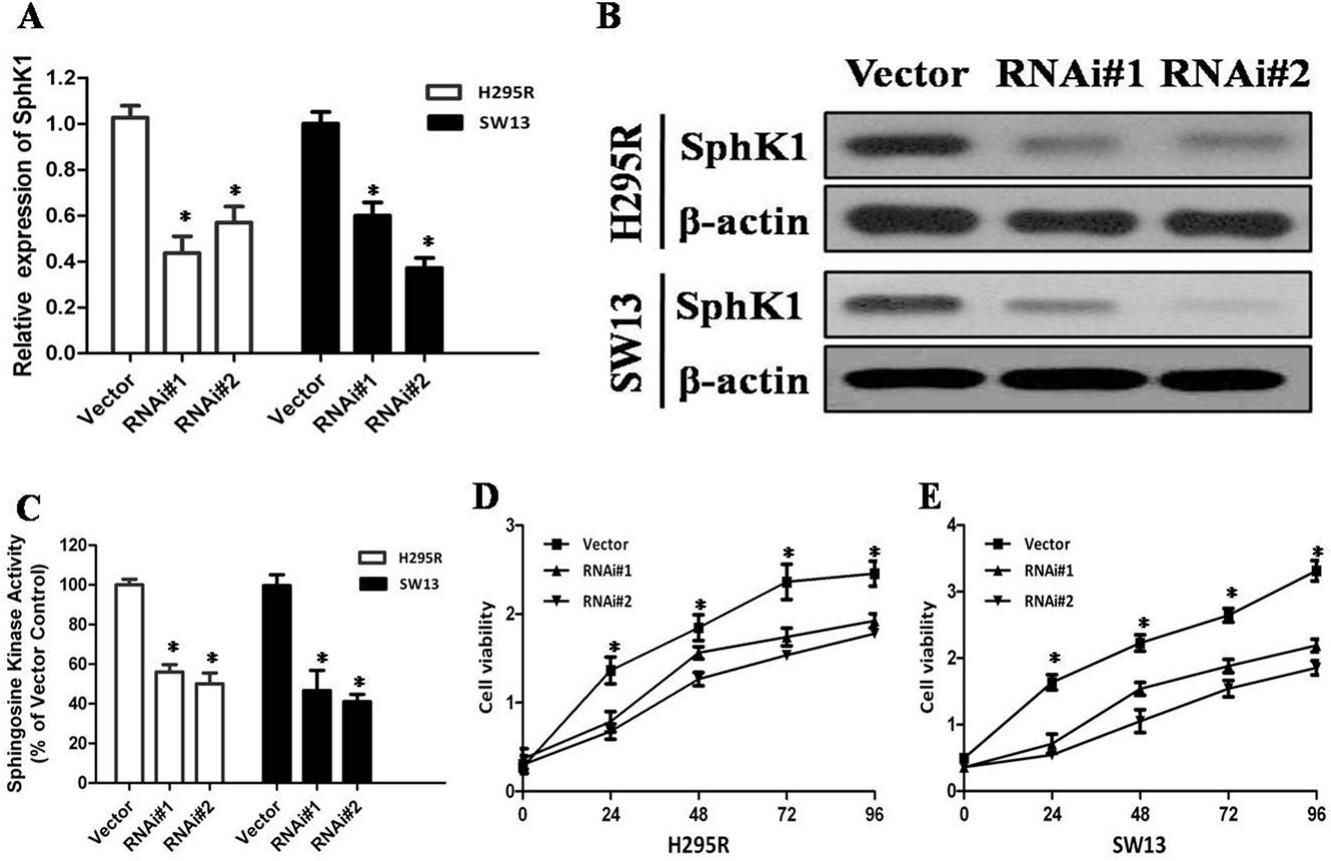
Depletion of SphK1 abrogates the proliferation of ACC cells (**A**) Knockdown of SphK1 in H295R and SW13 cell lines analyzed by real-time RT-PCR, SphK1 mRNA expression was normalized to GAPDH mRNA expression; (**B**) Knockdown of SphK1 in H295R and SW13 cell lines analyzed by western blot analysis, and β-actin was used as a loading control for western blot assays; (**C**) SphK1 enzymatic activity in SphK1-knocked down ACC cells was significantly decreased; (**D)** MTT assay was performed to evaluate the SphK1 on the proliferation of H295R at indicated time points; (**E**) MTT assay was performed to evaluate the SphK1 on the proliferation of SW13 at indicated time points.

**Figure 3:**
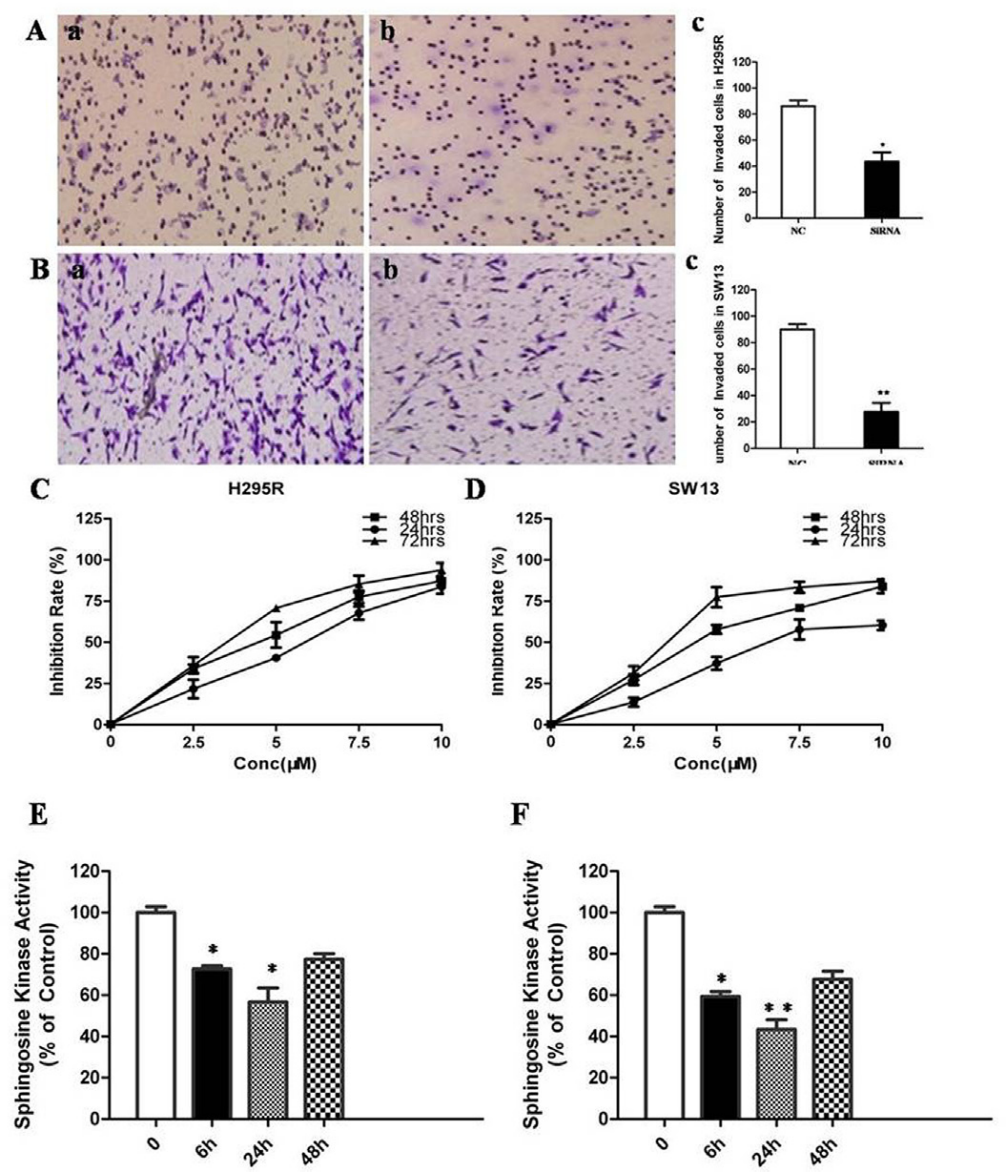
Inhibition of SphK1 surpresses the invasion and proliferation of ACC cells (**A**) Knockdown of SphK1 reduced invasion in H295R cell line; (**B**) Knockdown of SphK1 reduced invasion in SW13 cell line; (**C**) ACC cell lines were treated at the indicated concentrations for different time points, and the cell viability was analyzed by MTT assay. FTY720 induced cell death in H295R cells in a dose and time-dependent manner. (**D**) FTY720 induced cell death in SW13 cells in a dose and time-dependent manner; (**E**) FTY720 at a concentration of 10 μM significantly decreased SphK1 enzymatic activity in H295R cells; (**F**) FTY720 at a concentration of 10 μM significantly decreased SphK1 enzymatic activity in SW13 cells.

To confirm whether SphK1 regulates cell proliferation in ACC, we using siRNA to knockdown SphK1 expression in the two adrenal cancer cell lines, SW13 and H295R. Depletion of SphK1 by siRNA was verified at both RNA and protein levels (Figure 2A and 2B). Moreover, the enzymatic activities of SphK1 in SphK1-knocked down ACC cells were significantly decreased as compared with control (Figure 2C). MTT assay was performed to examine the effect of SphK1 on SW13 and H295R cells proliferation. As shown in Figure 2D and 2E, the results revealed that Sphk1-siRNA-transfected cells showed a significantly lower proliferation rate compared with the control groups, suggesting that SphK1 is involved in enhancing cell proliferation and might be associated with the transformed phenotype of ACC cells. To explore the effect of SphK1 on tumor metastasis, cellular invasiveness was analyzed by transwell assays, respectively. We found that knockdown of Sphk1 inhibited cell invasive ability in H295R and SW13 cells, suggesting that overexpression of Sphk1 in ACC could promote metastasis *in vitro* (Figure 3A and 3B).

### Sphingosine analogue FTY720 inhibits adrenocortical cancer cells proliferation via PI3K/Akt pathway

FTY720 is a sphingosine analogue and was previously shown to inhibit enzymatic activity of recombinant SphK1 [11]. H295R and SW13 cells were treated with 2.5, 5, 7.5, 10 μM of FTY720 for 24, 48 or 72 hrs, respectively. The results of MTT assay showed that FTY720 induced a dose and time-dependent decrease of cell viability in these two cell lines (Figure 3C and 3D), with IC50 values of 6.09 μM and 5.18 μM in SW13 and H295R cells at 48 h of treatment, respectively. FTY720 at a concentration of 10 μM could induce a rapid inhibition of SphK1 enzymatic activity in SW13 and H295R cells (−36 ± 3% and −42 ± 5% in SW13 and H295R cells, respectively, at 6 hours; Figure 3E and 3F).

**Figure 4:**
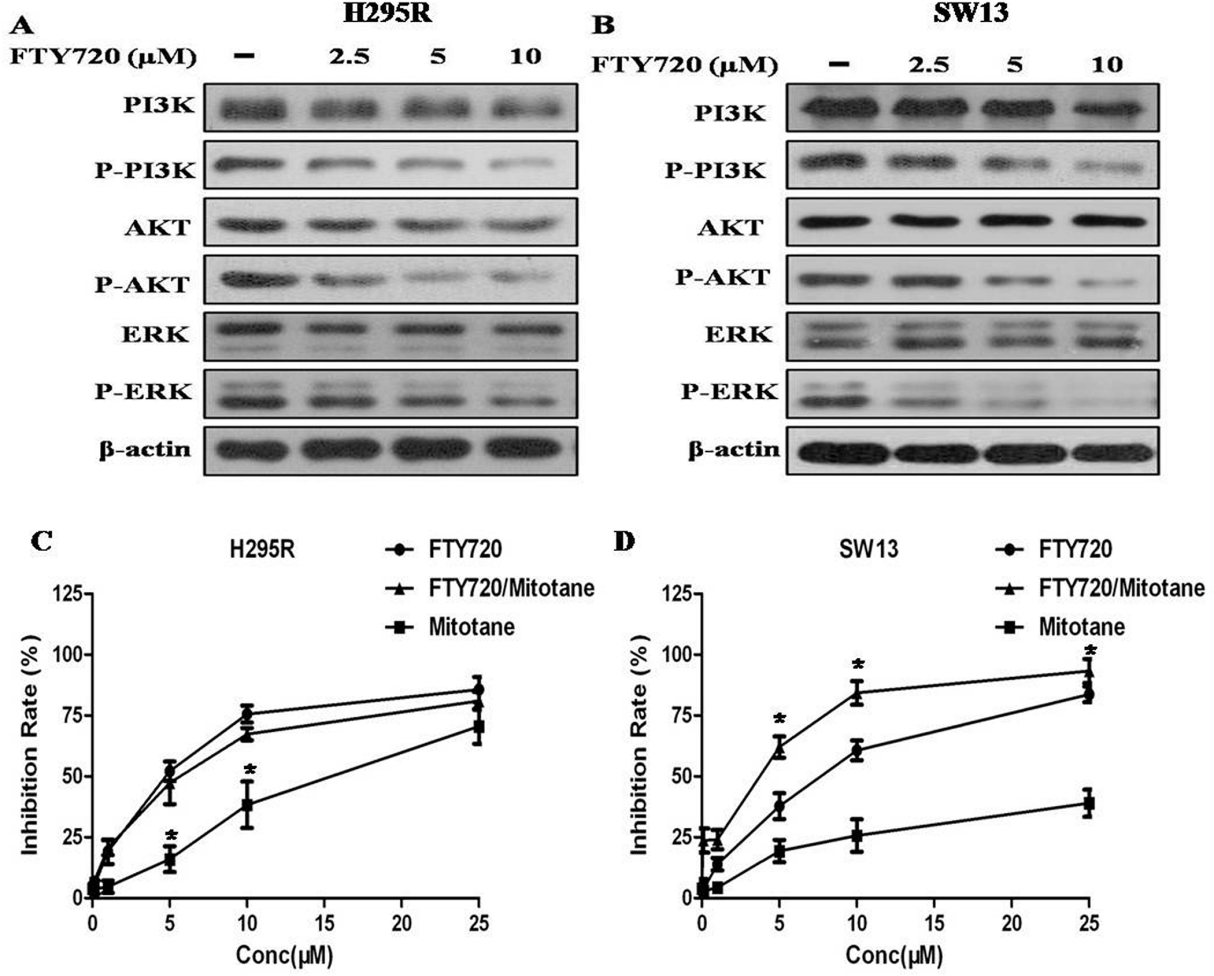
Effect of FTY720 on the activities of major signaling pathways, and effect of FTY720/mitotane combination on the proliferation of ACC cells (**A**) The antibodies against PI3K, phospho-PI3K^Y607^ (p-PI3K), AKT, phospho-AKT^S473^ (p-AKT), ERK, phospho-ERK ^pT202/pY204 + pT185/pY187^ (p-ERK) were used to determine the effect of FTY720 on the activities of PI3K/Akt and MAPK signaling. Western blotting analysis revealed that FTY720 significantly reduced the levels of p-PI3K, p-AKT and p-ERK in a dose dependently manner in H295R cells; (**B**) Western blotting analysis revealed that FTY720 significantly reduced the levels of p-PI3K, p-AKT and p-ERK in a dose dependently manner in SW13 cells; (**C**) Cell viability after single or combined mitotane and FTY720 treatment. Cytotoxic response to mitotane and the combination of mitotane and FTY720 in H295R and SW13 cell lines.; (**D**) The combination of mitotane and FTY720 showed a significant synergistic anti-proliferative effect (CI = 0.90 ± 0.08) in SW13 cells that potentiated the cytotoxic effect observed by using FTY720 alone.

To explore the mechanisms underlying the antitumor effects of FTY720, we investigated the effect of FTY720 on the activities of PI3K/AKT and MAPK signaling, which play an important role in cell proliferation and survival in cancer [12–13]. When the H295R and SW13 cells were treated with 2.5, 5, 10 μM of FTY720 for 48 h, and the results of western blot revealed that FTY720 significantly reduced the levels of p-PI3K, p-AKT and p-ERK, indicating that FTY720 specifically attenuated the PI3K/AKT and MAPK signaling pathways (Figure 4A and 4B).

As expected by the known profiles of mitotane-responsiveness of the two cell lines, mitotane determined a cytotoxic effect in H295R cells with a IC50 value of 18.8 μM (at 48 h of treatment), however, the IC50 value in SW13 cells is more than 50 μM (at 48 h of treatment) (Figure 4C and 4D). The combination of mitotane and FTY720 showed a significant synergistic anti-proliferative effect in SW13 cells (CI = 0.90 ± 0.08) that potentiated the cytotoxic effect observed by using FTY720 alone, but not in H295R cells.

### FTY720 induces apoptosis in ACC cells by inhibition of SphK1

**Figure 5:**
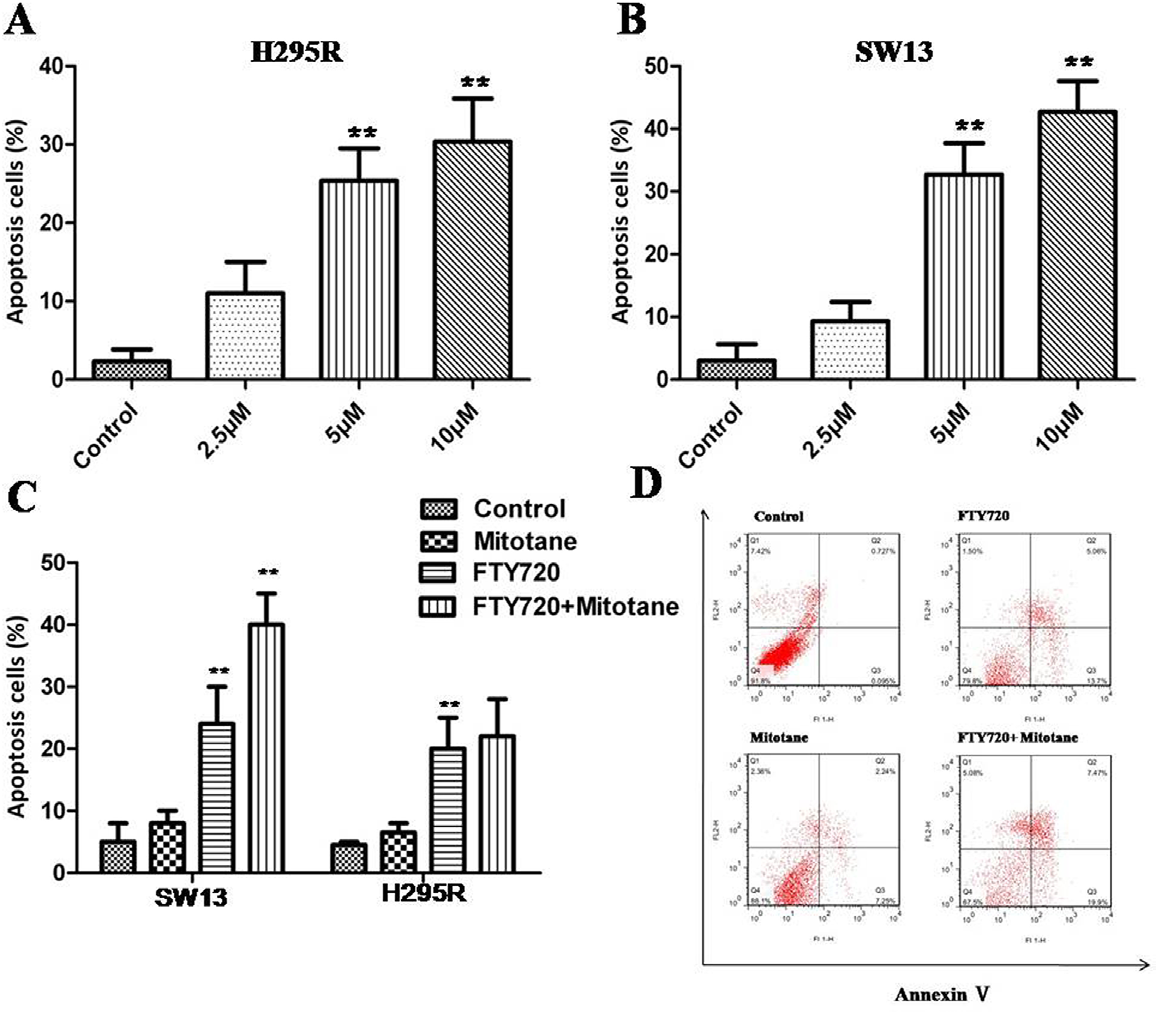
Induction of apoptosis by FTY720 in ACC cells Flow cytometry results of annexin V-PI stained ACC cells after exposure to different concentration of FTY720 for 24 h. (**A**) An increase in apoptotic cells following treatment with FTY720 is shown in H295R cells; (**B**) An increase in apoptotic cells following treatment with FTY720 is shown in SW13 cells; (**C**) The combination of FTY720 and mitotane had a significant influence on the apoptotic rate in SW13 cells, but there is no effect of the combination of FTY720 and mitotane on the apoptosis of H295R; (**D**) The flow cytometry profile represents SW13 cells with annexin V-FITC staining on the x-axis and PI on the y-axis. Dual staining of cells with annexin V-fluorescein isothiocyanate (FITC)/propidium iodide (PI) FITC and PI enabled categorization of cells into 4 regions. Region Q1 shows dead cells, Q2 shows late apoptotic cells, Q3 shows early apoptotic cells, and Q4 shows living cells. Q2 and Q3 are collectively called apoptotic cells.

Annexin V and PI staining results showed that with the increase of FTY720 concentration after 24 h, both in H295R and SW13 cells, the number of apoptotic cells increased obviously (*P* < 0.01; Figure 5A and 5B). However, Early and late apoptotic cell ratios were not modified by mitotane treatment neither in H295R nor in SW13 cells (*P* > 0.05, Figure 5C). Moreover, the combination of FTY720 and mitotane had a significant influence on the apoptotic rate in SW13 cells, but there is no obvious enhancement effect of the combination of FTY720 and mitotane on the apoptosis of H295R (Figure 5C and 5D).

### FTY720 inhibits xenograft tumor growth

**Figure 6:**
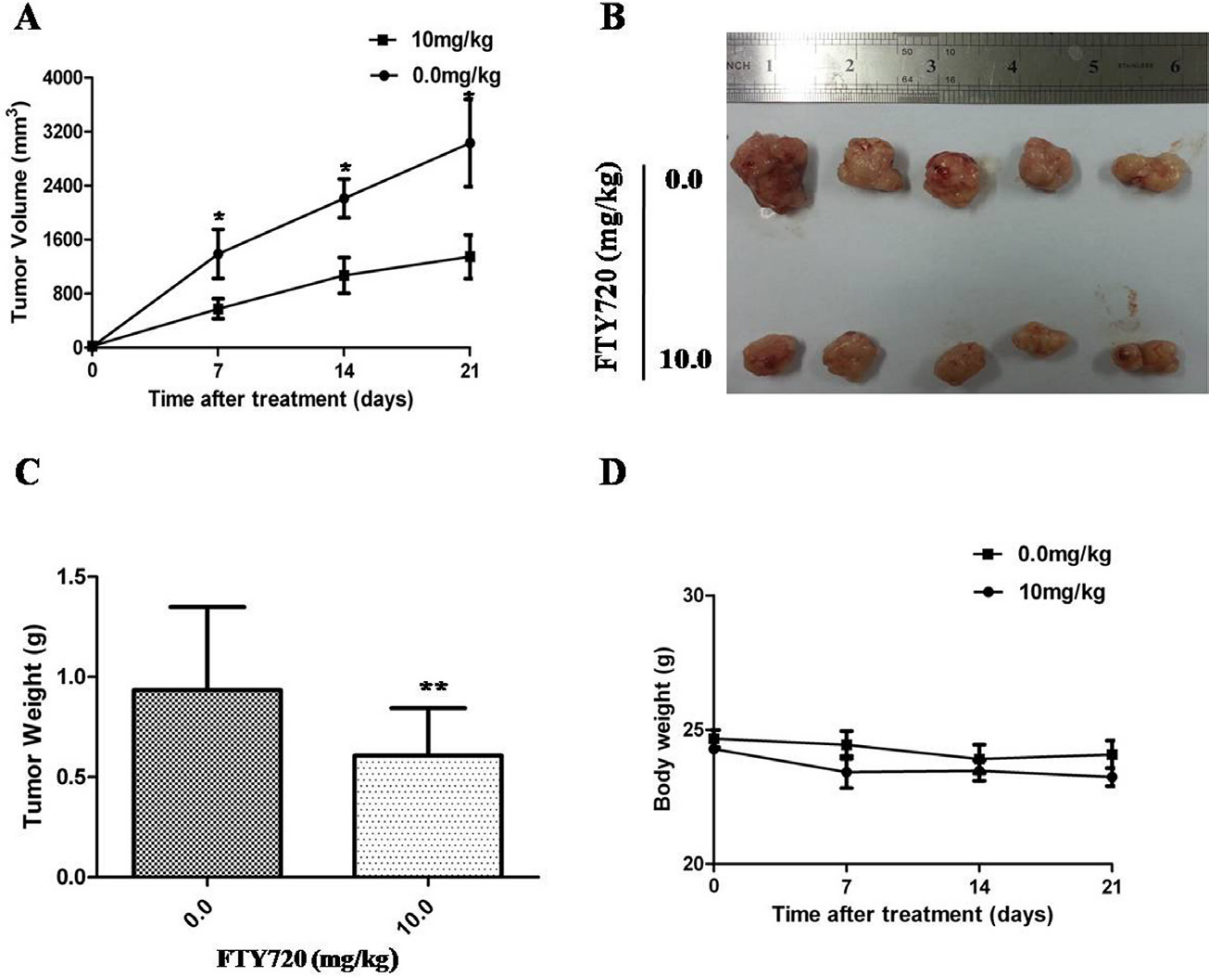
Inhibition of xenograft tumor growth by FTY720 (**A**) Time course of tumor growth, measured as tumor volume in each group at the indicated time of treatment with vehicle control or FTY720 (10 mg/kg; ip.). Data are presented as mean ± SD; (**B**) Representative picture of tumor growth in mice treated with the indicated concentration of FTY720 and vehicle control; (**C**) The bar graph represents mean of the tumor weight from FTY720-treated and control mice. Data are presented as mean ± SD. **, *P* < 0.01; (**D**) Compared with control group, the mean body weight was not significantly change in FTY720-treated group.

Given the potent inhibitory effects of FTY720 on ACC growth *in vitro*, it is believed that FTY720 has the potential to be highly effective in treating ACC *in vivo*. Thus, we sought to determine the inhibition effect of FTY720 on the tumor growth of H295R cell xenograft. When tumors reached 70–100 mm^3^, saline or FTY720 (10 mg/kg) was administered by IP injection per day for 10 days and tumor growth was observed for more 10 days after the termination of treatment. Our results found that H295R cell-derived xenograft tumors progressively grew in the control group, whereas the tumors were slow-growing in the FTY720-treated group. Tumor volume was significantly lower in FTY720-treated mice as compared with control mice (Figure 6A and 6B; *P* < 0.05). At the end of experiments, the tumors were isolated and weighted. Compared with control group, the mean tumor weight was significantly less in FTY720-treated group without affecting body weight obviously (*P* < 0.01; Figure 6C and 6D).

## DISCUSSION

In this study, we determined the critical roles of SphK1 in ACC pathogenesis and the underlying mechanisms. First, the SphK1 expression was overexpressed at the transcription level in ACC tissues, as we observed a significant increased SphK1 mRNA level in the ACC group. Second, the SphK1 protein level was upregulated in ACC group compared with the ACA tissues, suggesting a novel signaling pathway that contributes to ACC pathogenesis and aggressive biology. We also demonstrated that inhibiting SphK1 activity with specific SphK1 inhibitor or downregulating SphK1 with RNAi might represent a novel strategy for the treatment of ACC.

SphK1 is elevated in various types of cancers, functioning as an oncogene in tumorigenesis [14–15], however, little is known about the role of SphK1 in ACC progression. We found that SphK1 is upregulated in ACC and that high expression of SphK1 significantly associated with advanced stage and poorer overall survival in ACC patients. Numerous studies have shown that SphK1 is found to enhance the proliferation ability of cancer cells. Gucluler et al. showed that overexpression of SphK1 enhances tumor formation of human breast cancer MCF-7 cells in nude mice [16]. In contrast, inhibition of SphK1 could attenuate lung cancer cell growth *in vitro* as well as *in vivo* [17–18]. Consistent with previous studies, our results found that knockdown of SphK1 by siRNA markedly surpressed the ability of cell proliferation and invasion of two ACC cell lines, SW13 and H295R cells.

Recently, targeting the Sphk1 pathway with specific inhibitors or drugs is an appealing strategy for the development of effective cancer therapy [19–20]. A sphingosine analogue, dimethylsphingosine, was the first reported SphK inhibitor to surpress cancer cell growth [21]. FTY720 was originally developed as an immunosuppressive agent, which induces lymphopenia via an inhibition of lymphocytes' egress from lymphoid organs through its antagonist function on the lymphocytes' S1P receptors [22]. In addition, FTY720 could act as a SphK1 inhibitor to exhibit the antiproliferative effect in various cancers in a manner independent of S1P receptors [23–25]. In the present study, we demonstrated for the first time that FTY720 is able to inhibit ACC cell growth not only *in vitro*, but also *in vivo* in a xenograft model. In a mouse model of ACC, administration of FTY720 at 10 mg/kg/day inhibited tumor growth without causing detectable toxicity in vital organs. FTY720 also has a capacity to inhibit SphK1 enzymatic activity, suggesting that FTY720 is a SphK1 inhibitor and may be a new anticancer agent candidate for ACC.

Although the evidence has highlighted the potent antitumor effect of FTY720 on ACC, the molecular mechanism is still unclear. To explore the mechanisms underlying the antitumor effects of FTY720, we tested the effect of FTY720 on the MAPK and PI3K/AKT pathways, which have been demonstrated to be involved in tumor progression [26–27]. It is also well accepted that overexpression of SphK1 is involved in the activation of the PI3K/AKT and MAPK pathways [28–30]. It was consistent with our finding that FTY720 significantly inhibited the activity of the MAPK and PI3K/AKT pathways. FTY720 strongly inhibited phosphorylation of PI3K and AKT as well as ERK expression. Mitotane (o,p′-DDD) is the standard of care for treatment of ACC with specific adrenocortical activity [31]. Mitotane is effective at inducing a reduction of cell viability in H295R but not in SW13 cells. In this study, we investigated the effect of combining mitotane with FTY720 in ACC cells, and the results showed that the combination only resulted in a greater anti-proliferative effect over single agent treatment in SW13 cells, indicating that the combined therapy of SphK1 inhibition and mitotane may not be always favorable for ACC patients.

Accumulating evidences have indicated that upreguration of SphK1 could induce cancer cells resistance to apoptosis, and FTY720 has also been shown to be a potent apoptosis inducer in various cancer cells [10, 32]. In the present study, inhibition of SphK activity by FTY720 could induce apoptosis in ACC *in vitro*, and the combination of FTY720 and mitotane had a significant enhancement effect on the apoptotic rate in SW13 cells.

In conclusion, we showed that SphK1 is overexpressed in ACC and leads to increased cell proliferation and invasion, and impairment of apoptosis, oncogenic transformation. In addition, inhibition of SphK1, using both FTY720 and siRNA, could suppress ACC progression *in vitro* and *in vivo*, suggesting that SphK1 inhibition might represent new and potential strategies against human ACC.

## MATERIALS AND METHODS

### Patients and tissue samples

The use of pathology specimens of patient subjects as well as the review of all pertinent patient record was approved by institutional ethics review board and patients themselves. Clinical parameters, such as sex, age at diagnosis, date of surgery, tumor size, and results of hormone analysis, and in case of ACC, tumor stage according to the European Network for the Study of Adrenal Tumors (ENSAT) classification. Forty-six adrenocortical tumor (ACT) tissues, collected from surgical specimens of 46 sporadic ACT patients, were divided into two groups: adrenocortical adenomas (ACAs) (N = 22) and ACCs (N = 24). Besides, freshly frozen tissue samples were available from 20 ACT patients (10 ACCs and 10 ACAs). Samples were snap-frozen in liquid nitrogen immediately after surgery.

### Cell culture and chemical reagents

The adrenocortical tumor cell lines H295R and SW13 were supplied by the American Type Culture Collection (ATCC, Rockville, MD, USA). H295R cells were cultured in a 1:1 mixture of Dulbecco's Modified Eagle's Medium and Ham's F–12 Nutrient mixture (DMEM/F12) (Sigma, St. Louis, USA) supplemented with 1% L-glutamine (Sigma) and 2.5% of Nu-Serum (BD Biosciences, San Jose, CA) and enriched with 1% di ITS + Premix (BD Bioscience). SW13 cells were cultured in DMEM (Sigma) supplemented with 10% fetal bovine serum (Biowest, France) and 1% L-glutamine (Sigma). The cell lines were incubated in a humidified atmosphere of 5% CO_2_ in air at 37°C. Mitotane was purchased from Supelco and dissolved in 100% methanol (Sigma). FTY720 was obtained from Novartis. Compounds were dissolved at 10 mM in dimethyl sulfoxide (DMSO) at stock solutions and stored at −20°C.

### RNA extraction and quantitative RT-PCR

Total RNA was isolated from cultured cells and primary tumor samples using TRIZOL reagent (Invitrogen) and was converted into first-strand cDNA with the first-strand cDNA synthesis kit (Promega) according to the manufacturer's instructions. Quantitative RT-PCR was performed using SYBR Master Mix (Takara) on a LightCycler 480 System (Roche Applied Science). A human GAPDH gene was used as an endogenous control for sample normalization. Results were presented as the fold expression relative to that of GAPDH. PCR primers were as follows: and for human SphK1, forward 5′-CTTGCAGCTCTTCCGGAGTC-3′ and reverse 5′-GCTCAGTGAGCATCAGCGTG-3′; for human GAPDH, forward 5′-GACTCATGACCACAGTCCATGC-3′ and reverse 5′-AGAGGCAGGGATGATGTTCTG-3′.

### Western blot analysis

Western immunoblot analyses were performed with protein lysates obtained from snap-frozen tissue samples and cultured cells. Twenty micrograms of denatured total protein was subsequently applied to one end of 10% SDS polyacrylamide gels. The proteins on the gel were then transferred onto a nitrocellulose membrane (Millipore, Temecular, California, USA). The membranes were blocked and then incubated overnight at 4°C with primary antibodies: anti-SphK1, anti-PI3K, antiphospho-PI3K (Y607), anti-AKT, antiphospho-AKT (S473), anti-ERK, antiphospho-ERK (pT202/pY204 + pT185/pY187) and β-actin (Abcam, Cambridge, MA, USA). Membranes were washed three times and incubated with horseradish peroxidase-conjugated secondary antibodies (Abcam, Cambridge, MA, USA). Target protein bands were visualized using the enhanced chemiluminescence method. All western immunoblot analyses were performed three times.

### Immunohistochemistry (IHC)

Specimens were fixed in formalin and embedded in paraffin. A 4-μm section of each specimen was stained for stratifin. The primary antibody against SphK1 (Rabbit anti-Sphk1 polyclonal antibody, Abcam, Cambridge, MA, USA) was diluted (1:100) and added to the slides that were then incubated overnight at 4°C. The slides were followed by a PBS wash and incubated by anti-rabbit EnVisionTM kit (DAKO, USA) for 30 min at 37°C. The positive controls were gastric carcinoma with positive expressions of SphK1. PBS instead of primary antibodies was as negative control. The degree of immunostaining of SphK1 protein was examined and scored independently by two observers by combining both the proportion of positive staining tumor cells and the staining intensity. Scores representing the proportion of positively stained tumor cells was graded as: 0 (no positive tumor cells), 1 (< 10%), 2 (10–50%), or 3 (> 50%). The intensity of staining was determined as: 0 (no staining), 1 (weak staining, light yellow), 2 (moderate staining, yellow brown), or 3 (strong staining, brown). The staining index was calculated as the product of staining intensity and percentage of positive tumor cells, resulting in scores of 0, 1, 2, 3, 4, 6, and 9. Specimens with staining index score of more than or equal to four was considered as high expression, and less than or equal to three as low expression.

### Knockdown of SphK1 by siRNA transfection

Sequence-specific siRNA against SphK1 was used to silence SphK1 expression (Santa Cruz Biotechnology, USA). The siRNA sense sequence were 5′-GGGCAA GGCCUUGCAGCUCd(TT)-3′ and 5′-GAGCUGCAAGGCC UUGCCCd(TT)-3′ (for SphK1) or scrambled siRNA 5′-UUCUC CGAACGUGUCACGUd(TT)-3′ and 5′-ACGUGACACGU UCGGAGAAd(TT). Cells were seeded into six-well plates at a density of 3 × 10^4^ cells per well and transfected with siRNA (50–100 nM) using the lipofectamine RNAiMAX transfection reagent (Invitrogen, Carlsbad, CA, USA), following the manufacturer's instructions. After 48 h, SphK1 expression levels were detected by RT-PCR and/or Western blot.

### Sphingosine kinase activity assay

The activity of sphingosine kinase was quantified by using a commercial Sphingosine Kinase Activity Assay kit (Echelon Biosciences, Salt Lake City, USA) as the manufacturer instructed.

### Cell proliferation assay

The effects of SphK1 siRNA and FTY720 on ACC cells survival were determined by MTT assay. Cells were seeded into 96-well plates (5 × 10^3^ cells/well) and cultured for 120 h. After treatments, cells were incubated with MTT (Sigma-Aldrich, St. Louis, MO, 20 μl/well) at 37°C for 4 h, and then 200 μl DMSO was added into each well. Cells were subjected to absorbance reading at 570 nm using a 96-well microplate reader. Drug interaction was assessed using the combination index (CI). Values of CI < 1, equal to 1, and > 1 indicate synergistic, additive and antagonistic effects, respectively.

### Flow cytometric analysis

Apoptotic cell death was identified by double supravital staining with recombinant FITC (fluorescein isothiocyanate) conjugated Annexin V and propidium iodide (PI), using the Annexin V-FITC Apoptosis Detection kit (Becton-Dickinson, NJ, USA) according to the manufacturer's instructions.

### Cell invasion assay

The extent of cell invasion was assessed using the BD BioCoatTM MatrigelTM Invasion Chamber (BD Biosciences, Bedford, MA), according to the manufacturer's protocol.

### Tumor xenograft study

Animal studies were performed in accordance to an institutionally approved protocol of Shanghai Jiao Tong University. Tumor xenografts were established by sc injection of 2 × 10^6^ H295R cells into flank regions of 4- to 6-wk-old female athymic mice. When the tumor reached 70–100 mm^3^ in volume, intraperitoneal injections of saline or FTY720 (10 mg/kg) were administered. Mice were checked weekly, and tumor nodules were measured with a caliper. The mice were sacrificed and tumors were excised and weighed at the end of *in vivo* experiments.

### Statistical analysis

All statistical analyses were carried out using the SPSS 10.0 statistical software package. The χ^2^ test was used to analyze the relationship between SphK1 expression and the clinicopathologic characteristics. Survival curves were plotted by the Kaplan-Meier method and compared by the log-rank test. In all cases, *P* < 0.05 was considered statistically significant.
